# Exploring synthetic lethal network for the precision treatment of clear cell renal cell carcinoma

**DOI:** 10.1038/s41598-022-16657-7

**Published:** 2022-08-02

**Authors:** Zhicheng Liu, Dongxu Lin, Yi Zhou, Linmeng Zhang, Chen Yang, Bin Guo, Feng Xia, Yan Li, Danyang Chen, Cun Wang, Zhong Chen, Chao Leng, Zhenyu Xiao

**Affiliations:** 1 Department of Hepatic Surgery Center, Tongji Hospital, Tongji Medical College, Huazhong University of Science and Technology, Wuhan, 430030 Hubei China; 2 Department and Institute of Urology, Tongji Hospital, Tongji Medical College, Huazhong University of Science and Technology, Wuhan, 430030 Hubei China; 3 State Key Laboratory of Oncogenes and Related Genes, Shanghai Cancer Institute, Renji Hospital, Shanghai Jiao Tong University School of Medicine, Shanghai, 200030 China; 4 Department of Immunology, Zhongshan School of Medicine, Sun Yat-Sen University, Guangzhou, 510080 Guangdong China; 5 Department of Neurology, Tongji Hospital, Tongji Medical College, Huazhong University of Science and Technology, Wuhan, 430030 Hubei China

**Keywords:** Cancer genetics, Computational biology and bioinformatics

## Abstract

The emerging targeted therapies have revolutionized the treatment of advanced clear cell renal cell carcinoma (ccRCC) over the past 15 years. Nevertheless, lack of personalized treatment limits the development of effective clinical guidelines and improvement of patient prognosis. In this study, large-scale genomic profiles from ccRCC cohorts were explored for integrative analysis. A credible method was developed to identify synthetic lethality (SL) pairs and a list of 72 candidate pairs was determined, which might be utilized to selectively eliminate tumors with genetic aberrations using SL partners of specific mutations. Further analysis identified *BRD4* and *PRKDC* as novel medical targets for patients with *BAP1* mutations. After mapping these target genes to the comprehensive drug datasets, two agents (BI-2536 and PI-103) were found to have considerable therapeutic potentials in the *BAP1* mutant tumors. Overall, our findings provided insight into the overview of ccRCC mutation patterns and offered novel opportunities for improving individualized cancer treatment.

## Introduction

Renal cell carcinoma (RCC) is one of the most common malignancies in the genitourinary system. A recent study showed that 431,288 new cases and 179,368 deaths of RCC occurred in 2020^1^. Approximately 70% of renal cancers are localized stage, indicating the possibility of complete tumor excision by radical nephrectomy^2,3^. Clear cell renal cell carcinoma (ccRCC) is the most prevalent subtype, accounting for more than 70% of all RCC^4^. Although most ccRCCs are effectively treated, by surgery or ablation when diagnosed early, the distant metastasis rate is up to 33% after treatment^5^. Considering the poor prognosis of ccRCC patients, more efforts are required in developing optimal adjuvant or targeted therapies.

With the rapid development of genome sequencing and the availability of tremendous genomic information on carcinoma, the significant role of driver mutation (DM) in the occurrence and development of renal cancer was proved^6^. And genetically targeted drugs have been successfully used in patients with gene mutations. VEGFR inhibitor sunitinib and mTOR signaling inhibitor everolimus are frequently used for renal cancers. Nonetheless, many patients still suffer from tumor recurrence due to drug resistance. The SL strategy provides a promising approach for the treatment of renal cancers, and a robust evident is the effective and tailored anti-cancer compounds, such as poly (ADP-ribose) polymerase (PARP) inhibitor olaparib. Briefly, the simultaneous mutation of a specific gene pair causes tumor cell death, and the functional loss of either one has little effect on cell survival. Since many challenges are faced in pharmacologically rescuing the function of mutated genes such as von Hippel-Lindau (*VHL*) and BRCA1 associated protein 1 (*BAP1*), the drugs targeting a second-site of SL pairs are considered an alternative method in treating patients with gene mutations. Harnessing this concept, current investigations have focused on identifying SL gene pairs associated with *VHL*-hypoxia-inducible factor (*HIF*) signaling^7–9^. To find more SL gene pairs with therapeutic potentials, it is necessary to expand the process of screening molecular candidates.

Previous studies proposed various algorithms to identify SL gene pairs, such as *DAISY*^9^ and *MiSL*^10^. Nevertheless, such procedures mainly use non-specific inference for pan-cancer analysis, which could be unsuitable for renal cancers with specific mutation patterns. Therefore, we aimed to conduct a comprehensive literature review to search for publicly available data on ccRCC in this study. Then a novel strategy of SL interaction analysis was applied to identify the potential SL gene-partners of driver genes in ccRCC. The paired genes with therapeutic implications will be identified after filtering out the candidate SL pairs, and compounds collected from multiple drug databases will be matched to identify potential therapeutic candidates for tumor patients. Generally, our findings may provide comprehensive insight into the mutation pattern of ccRCC, and new opportunities for exploring highly specific therapeutic targets for renal tumors.

## Result

### Overview of the SL interaction analysis

**Figure 1:**
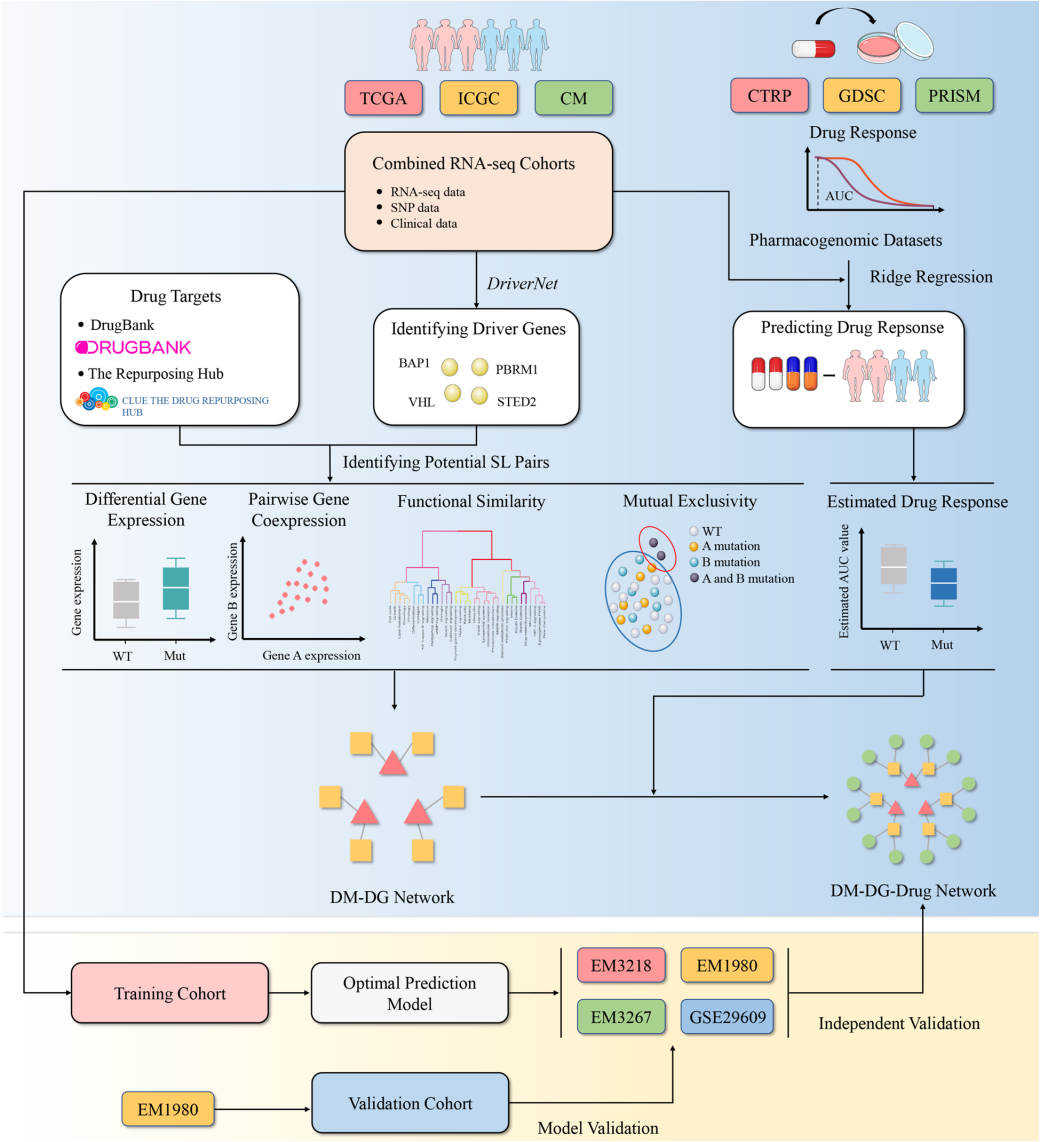
Flow chart of identification of potential synthetic lethal interactions and construction of DM-DG-drug networks.

A total of 1174 ccRCC transcriptome profiles together with clinical information were collected from numerous publicly available cohorts, including the Cancer Genome Atlas Kidney Renal Clear Cell Carcinoma (TCGA-KIRC)^11^, renal cell cancer-EU (RECA-EU), CheckMate 009 (CM-009)^12^, CheckMate 010 (CM-010)^13^, CheckMate 025 (CM-025)^14^, E-MTAB-1980, E-MTAB-3218, E-MTAB-3267 and GSE29609. Of these patients, 928 were from RNA sequencing data, which were used for further SL framework construction, and 246 were from microarray data, which were considered external validation for evaluating the result of the DM-druggable gene (DG)-drug network. A schematic diagram of the procedures of SL inference and the overall study design are presented in Fig. 1.

The SL interaction analysis is a computational pipeline evaluation for identifying candidate SL interactions based on the experiences from several previous researches, such as *DAISY*^9^, *MiSL*^10^ and *SELECT*^15^. This analysis consists of four statistical inference procedures:

1. Differential gene expression: The procedure exploited gene expression and somatic alterations of the inputting tumor samples to discover potential SL gene pairs under the assumption that carcinoma cells may increase the expression of its SL partners as a compensatory mechanism when a driver gene loses its function due to the mutation. Differential expression analysis was conducted using Wilcoxon rank-sum test between the samples with and without DMs, and only target genes with higher expressions in the mutated samples were saved as potential SL partners of corresponding DMs.
2. Pairwise gene co-expression: The procedure tended to select gene pairs which could have similar functions of cell metabolism and growth, and be likely co-expressed in the para-carcinoma normal tissues with the notion that there is often an intensive relationship between both genes of the SL pair. Gene pairs presenting significant correlations (Spearman correlation coefficient > 0.1 and *P adjust* < 0.05) were considered as SL candidate pairs.
3. Functional similarity: The procedure aimed to filter out the gene pairs with high semantic similarity, motivated by the assumption that the SL partners tend to engage in closely related biological processes. And accordingly, their locations in Gene Ontology (GO) topological structure should be close. The functional similarity score (FSS), which was defined as the geometric mean of semantic similarities of molecular function (MF) and cellular component (CC), ranged from 0 to 1. And FSS ≤ 0.45 between gene pairs were considered to have no significant functional similarity and thus they were excluded from the candidate SL pairs.
4. Mutual exclusivity: The procedure selected those gene pairs in which the incidence of simultaneous mutation was significantly lower than common gene pairs, based on the concept that simultaneous mutation of two genes in an SL pair would affect the cellular process and cause tumor cell death. The gene pairs with the *P adjust* < 0.15 were considered as potential SL pairs.

Those candidate pairs passing the requirements of all the four procedures composed the final output set of candidate SL pairs and were subsequently used for constructing the DM-DG-drug network.

### Detection of driver genes in ccRCC

**Figure 2:**
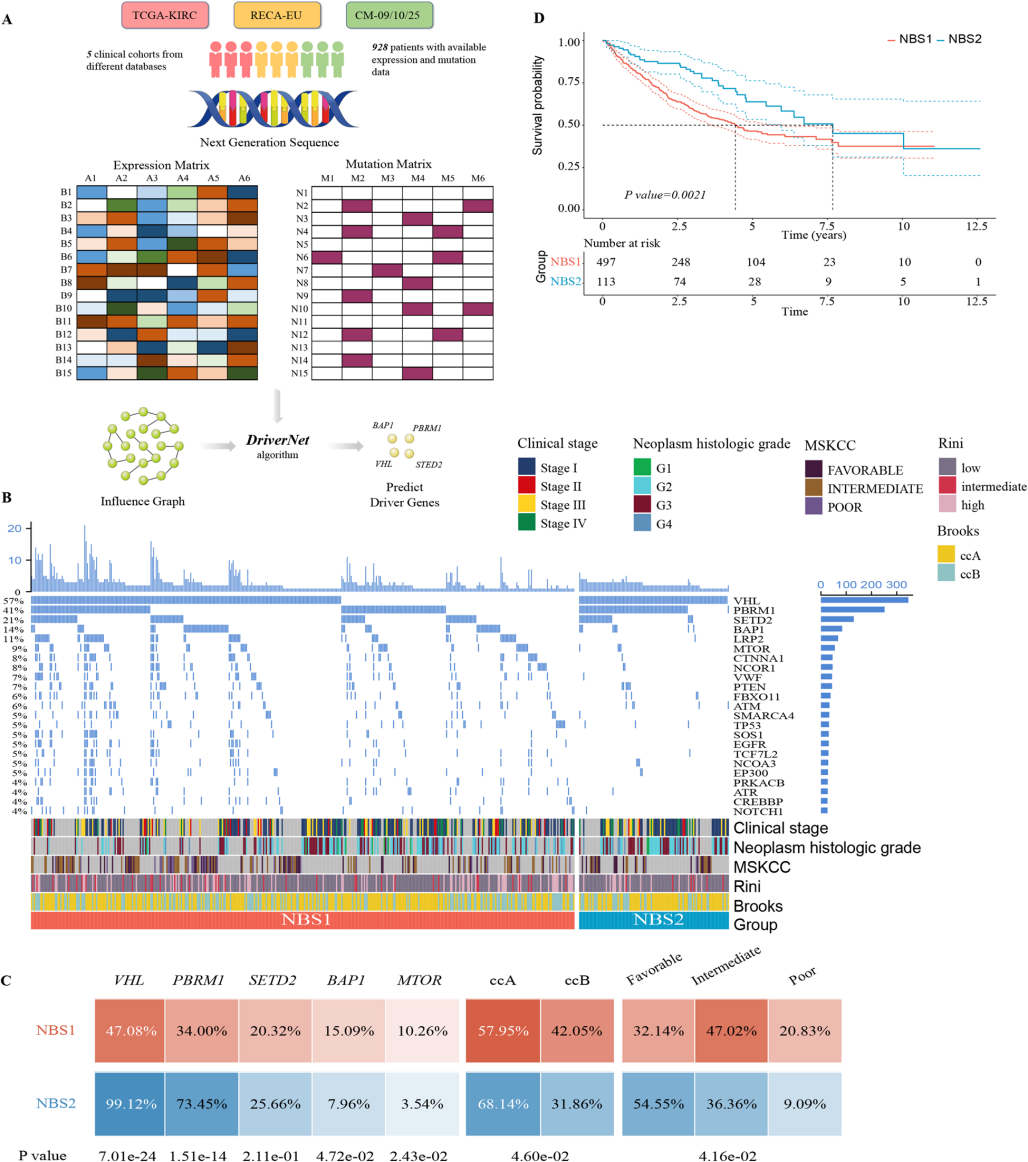
Identifying driver genes and subclass characteristics in clear cell renal cell carcinoma. (**A**). Overview of driver genes identification via *DriverNet* analysis in clinical cohorts. (**B**) The mutation profiles of subclasses classified by network-based stratification (NBS). Characteristics of clinical stage, histological grade, previously reported transcriptome-based molecular subclasses (MSKCC, Rini and Brooks) between two subclasses were presented simultaneously. (**C**) Difference in mutation frequency of driver genes, molecular characteristics stratified by Brooks and MSKCC between two subclasses. Fisher’s exact tests were applied to compared the statistical differences. (**D**) Kaplan–Meier survival curve of two subclasses. Statistical difference was calculated by log-rank test.

The current consensus on tumor development and progression is that only a few mutational events affecting driver genes were determined to be the origin of malignancy, which confers selective growth advantage to the tumor cell. Compared with traditional chemicals, small molecular compounds targeting DMs have the advantage of avoiding impairment of normal tissue, and thus screenings on these DMs are more likely to identify clinically significant targets. In this study, the DriverNet algorithm was applied to identify candidate drivers in the most comprehensive metadata set of ccRCC currently, which contained 610 patients from five clinical cohorts with both available expression and mutation data (Fig. 2A). A total of 36 candidate genes had been yielded with the *P adjust* < 0.1 and mutation frequency beyond the mean (Supplementary Table 1). Notably, due to the limitations of the influence graph derived from the Reactome functional interactions, *SETD2* which was confirmed as a driver gene in previous studies^11,16,17^ was added to our prediction model to generate more reliable results. Of these genes, 25 genes (67.6%) that demonstrated the reliability of our prediction have been reported by at least one previous research and were then taken as robust drivers of ccRCC for subsequent analysis.

To explore the clinical implications of DMs in ccRCC, the network-based stratification (NBS) algorithm was applied to stratify patients into different subtypes utilizing their mutation profiles. According to the outcome of cophenetic correlation coefficients, 610 patients were assigned into two groups (Supplementary Fig. 1A,B). The result indicated that each group had distinguishing mutation features (Fig. 2B). The NBS2 contained a higher proportion of common DMs, including *VHL*, *PBRM1* and *SETD2*, while the NBS1 consisted of a high frequency of *BAP1* and increased mutational burden (Fig. 2C and Supplementary Fig. 1C). Additionally, we analyzed the relationship between the NBS classification and the previously reported RCC molecular subgroups, including Rini’s (Low–High recurrence score group)^18^, Brooks’ (ccA-ccB group)^19^ and Motzer’s (Poor-Favorable risk group)^20^. The NBS1 was positively associated with Rini’s high recurrence score group (*P* = 0.1713), Brooks’ ccB group (*P* = 0.046) and Motzer ‘s poor risk group (*P* = 0.0416), while the NBS2 exhibited opposite patterns (Fig. 2C and Supplementary Table 2). Subsequently, the correlation between NBS classification and clinical characteristics, containing the clinical stage, pathological stage and survival time, was investigated using the combined cohort. A significant difference in survival outcome was found between the NBS groups, in which the NBS2 exhibited a better prognosis than the NBS1(*P* = 0.0021) (Fig. 2D). However, other clinical characteristics were weakly correlated with the NBS classification (Supplementary Table 2). Taking together, the NBS classification provided a novel insight into the DM-based clinical subclasses of ccRCC patients and enhanced our understanding of the crucial role of driver genes played in tumorigenesis and progression.

### Selection of druggable genes

The SL candidates of DMs were derived by leveraging the computational pipeline while encountering another problem that not all identified partners of DMs could be targeted when performing genome-wide scanning for potential SL partners. Therefore, to infer statistically significant SL partners that could be targeted by conventional chemical agents, a list of 4465 DGs was compiled from the current public pharmacological databases and considered as the input genes for SL analysis. Of these DGs, only 1981 target genes were used for constructing the DM-DG network due to low expression of some DGs after removing batch effects.

### Inference of driver mutation-druggable gene interactions

**Figure 3:**
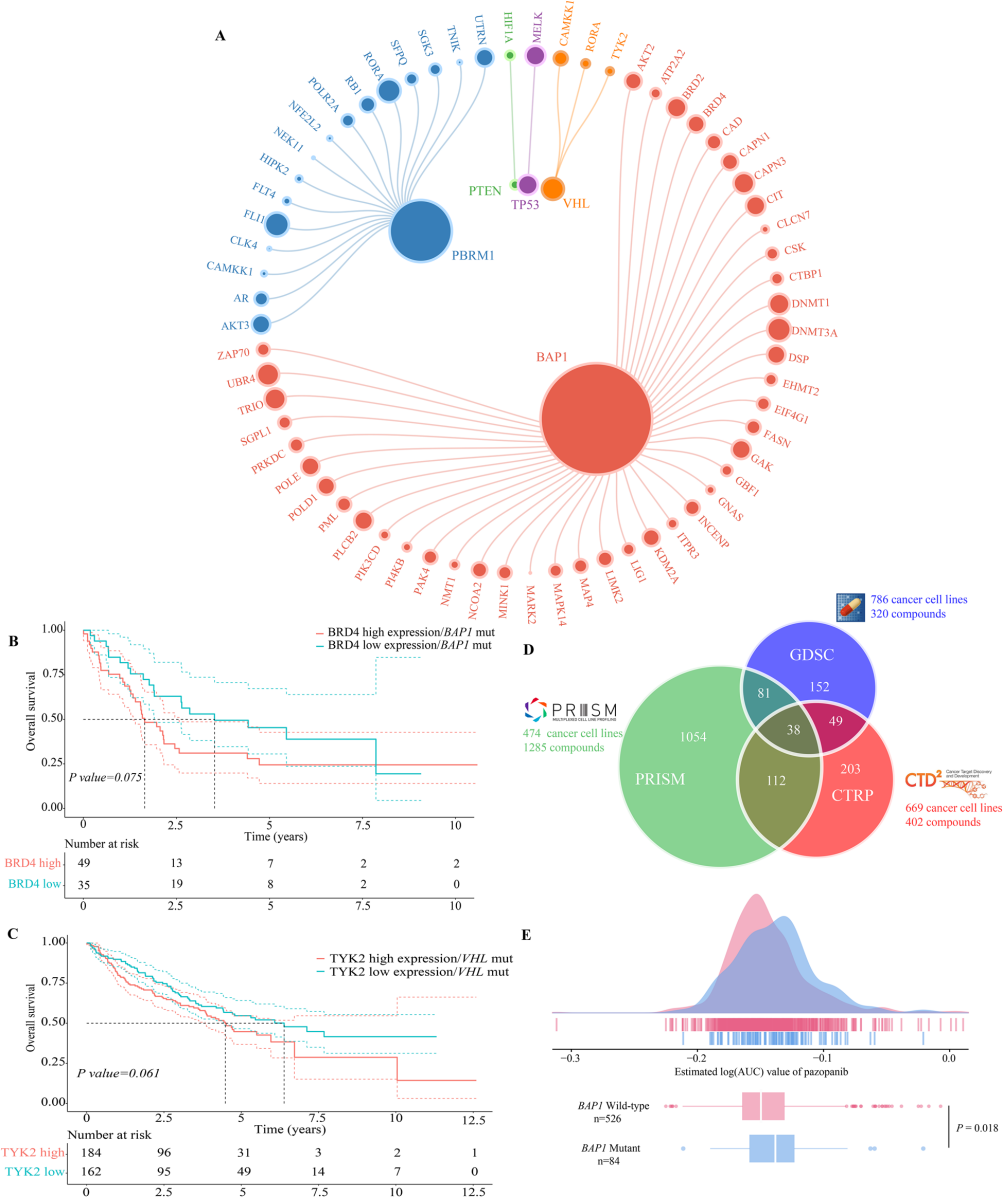
Exploring feasibility of druggable genes in treating driver mutation-specific clear cell renal cell carcinoma patients. (**A**) The bipartite network of representative DM-DG interactions. (**B**) Overall survival of distinct *BRD4* expression profiles in *BAP1* mutated patients. (**C**) Overall survival of distinct *TYK2* expression profiles in *VHL* mutated patients. (**D**) The venn graph for summarizing the available cancer cell lines and compounds in CTRP, PRISM and GDSC pharmacogenomic datasets. (**E**) Comparing estimated drug sensitivity (LogAUC) of pazopanib between *BAP1* mutated and wild-type samples.

Based on the selected 25 DMs and 1981 DGs, the SL interaction analysis was conducted to infer the DM-DG pairs which met the corresponding criteria. In total, 72 DM-DG pairs (containing 69 unique drug targets) passed all the screening procedures and thus considered SL candidates for ccRCC (Fig. 3A). Additionally, the rank aggregation analysis was performed to integrate the results of each procedure in SL interaction to obtain a robust ranking for the 72 DM-DG pairs. Accordingly, the ranks of candidate pairs were based on FS scores (functional similarity), fold change values (differential expression), correlation coefficients (pairwise co-expression), and *P adjust* (mutual exclusivity). Then, the Stuart method was applied to integrate all the rankings and calculated the rank aggregation score (RAS) for each DM-DG pair (Supplementary Table 3).

To validate whether the DM-DG pair exhibited SL interaction, we performed univariate survival analysis between DG expression in patients with specific DM and progression-free survival (PFS) using the Cox proportional hazards regression model. Partly significant DGs were associated with the shorter recurrence time (HR > 1) among patients with relevant DMs (Supplementary Table 4). Additionally, the Kaplan–Meier analysis was conducted to reveal the clinical relationship between PFS and the status of DG in patients with corresponding DM. Specifically, we mainly defined the functional status of one gene by dividing expression data into active (> median) and inactive groups (< median) for lacking aberration situation of DGs (Fig. 3B,C). As depicted from the figure, the *BRD4* and *TYK2* inactive groups had significant survival advantage in ccRCC patients with the *BAP1* and *VHL* mutations, compared with the active groups. These survival data-based analyses demonstrated that these DM-DG pairs have crucial clinical effects and were well compatible with their roles as SL candidates.

### Estimation of drug response in clinical samples

Three pharmacogenomic datasets described in the Materials and Methods section, containing drug sensitivity data and gene expression profiles from multiple cancer cell lines (CCLs), were utilized to construct the drug prediction model. The chemical compounds with NAs in more than 20% of the samples and CCLs derived from hematopoietic and lymphoid tissue were excluded to achieve the prediction result precisely. After removing the duplicated or invalid compounds, 1801 compounds were found in total. Of these, 669 CCLs with 402 compounds in the Cancer Therapeutics Response Portal (CTRP) dataset, 474 CCLs with 1,285 compounds in the PRISM dataset and 786 CCLs with 320 compounds in the Genomics of Drug Sensitivity in Cancer (GDSC) dataset were used for subsequent drug prediction analysis (Fig. 3D). The ridge regression model located in the package *pRRophetic* was applied to perform the drug response prediction for the clinical samples based on their expression profiles, and the estimated area under curve (AUC) value of each compound among clinical samples was used as an evaluation indicator for the drug sensitivity.

Before proceeding further, the results of drug response estimation were validated computationally. Pazopanib, an oral small-molecule multi-kinase inhibitor for the treatment of advanced renal cell carcinoma, was used to evaluate whether the estimated drug sensitivity was consistent with its clinical efficacy. A retrospective cohort study found that the mutation status of *BAP1* had independent prognostic value in advanced RCC patients treated with first-line tyrosine kinase inhibitors^21^. Compared with wild-type (WT) patients, those patients harboring the *BAP1* mutation performed worse outcomes from pazopanib treatment, with the unfavorable PFS and overall survival (OS). Therefore, patients from the combined RNA-seq cohort were categorized into the two groups according to their alteration statuses of *BAP1* (altered versus unaltered: 84 versus 526). The Wilcoxon rank-sum test was used to compare the estimated AUC values of pazopanib between the two groups, and the result suggested that a significantly higher value of patients with mutant *BAP1* than WT (*P* = 0.018) (Fig. 3E), consistent with the clinical behavior of pazopanib.

### Constructing prediction model of *BAP1* mutation

On the basis of the combined RNA-seq cohort, the elastic net (EN) algorithm described in the Materials and Methods section was utilized to construct a robust model for predicting *BAP1* mutation status. The differentially expressed genes between the *BAP1* mutant and WT samples contributed to this prediction model. Therefore, the *limma* package was applied to investigate the expression difference of these samples and differential genes were defined when *P adjust* < 0.05 and absolute log2 fold change (FC) > 1.

Survival analysis on 1,207 patients with prognostic and mutation data was conducted to investigate whether the functional status of the *BAP1* was associated with the survival outcome of cancer patients. A significant prognostic difference between the two groups was identified, with longer median survival time (MST) in WT patients (MST = 6.16 years, 95% confidence interval [CI]: 5.31–7.95 years) than in BAP1 mutant patients (MST = 2.46 years, 95%CI: 2.00–3.52 years), which was consistent with the results of the MSKCC and the TCGA-KIRC cohorts (Supplementary Fig. 2).

**Figure 4:**
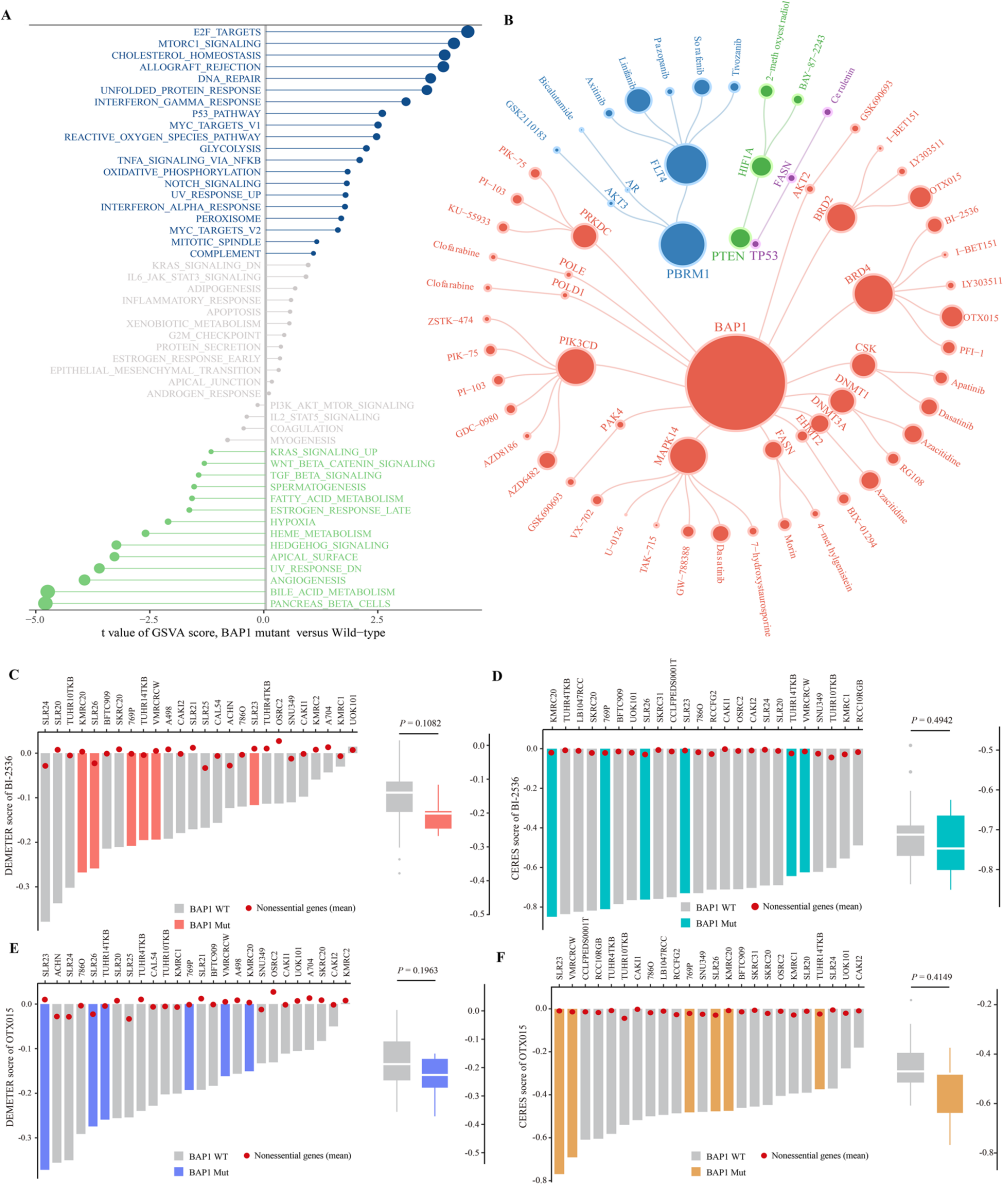
Determining sensitivities of identified drugs on renal cancer cell lines. (**A**) Gene set enrichment analysis between *BAP1* mutated and wild-type groups. Blue dots indicate *BAP1* mutant-enriched pathway, while red dots indicate wild type-associated pathways. (**B**) The bipartite network of representative TSG-DT-drug interactions. (**C**) The DEMETER scores derived from RNAi screens of BI-2536 across 24 kidney CCLs. (**D**) The CERES scores derived from CRISPR knockout screens of BI-2536 across 26 kidney CCLs. (**E**) The DEMETER scores derived from RNAi screens of OTX015 across 24 kidney CCLs. (**F**) The CERES scores derived from CRISPR knockout screens of OTX015 across 26 kidney CCLs.

The enrichment analysis was performed using R package *GSVA* to characterize the biological processes affected by the BAP1 mutation. The result showed that the up-regulated genes in the *BAP1* mutant group were enriched in multiple carcinogenesis associated pathways, such as E2F targets, MTORC1 signaling and DNA repair, while the up-regulated genes in the WT group were enriched in metabolism-associated pathways, such as pancreatic beta cells and bile acid metabolism (Fig. 4A and Supplementary Table 5).

Based on the *BAP1* mutation prediction model, the prediction accuracy was 93.1% in the training cohort (combined RNA-seq cohort) and 84.2% in the independent validation cohort (E-MTAB-1980) (Supplementary Fig. 3A,B). To evaluate the ability of the prediction model, the receiver operating characteristic (ROC) curve was used using R package *pROC*, and a higher AUC indicated a preferable performance of the model. The AUC of this prediction model was 0.956 in the training cohort and 0.895 in the validation cohort (Supplementary Fig. 3C,D), suggesting that this model was efficient and robust enough for predicting the *BAP1* alteration in other transcriptomic cohorts. Therefore, this model was used to identify the estimated *BAP1* mutant samples from the combined microarray cohort (E-MTAB-3267, E-MTAB-3218, E-MTAB-1980 and GSE29609).

### Identification of therapeutic candidates for the *BAP1* mutant ccRCC

According to the target annotation, 167 associated drugs were retained after mapping drugs to 69 unique targets in the DM-DG pairs. The differential drug response analyses between the WT and mutant patients were conducted to further connect DMs with these DG-associated drugs. Compared with WT samples, only drugs with significantly lower estimated AUC values in the mutated samples (logFC < 0 and *P* value < 0.05) were considered SL-associated drugs. There remained 149 DM-drug pairs and 49 DM-DG pairs met the screening requirements, which were then visualized in a DM-DG-drug network (Fig. 4B and Supplementary Table 6). Among the final candidate SL pairs, the number of *BAP1* mutant gene pairs was far more than other DM-DG pairs, which provided more potential therapeutic agents for this kind of patients. Since *BAP1* mutated tumors were significantly associated with worse overall survival than tumors without mutated *BAP1*^6^, it was essential to investigate the specialized therapeutic agents for the *BAP1* mutant ccRCC. Accordingly, the *BAP1* mutation was selected for further investigations regarding its therapeutic potential in renal cancers.

**Figure 5:**
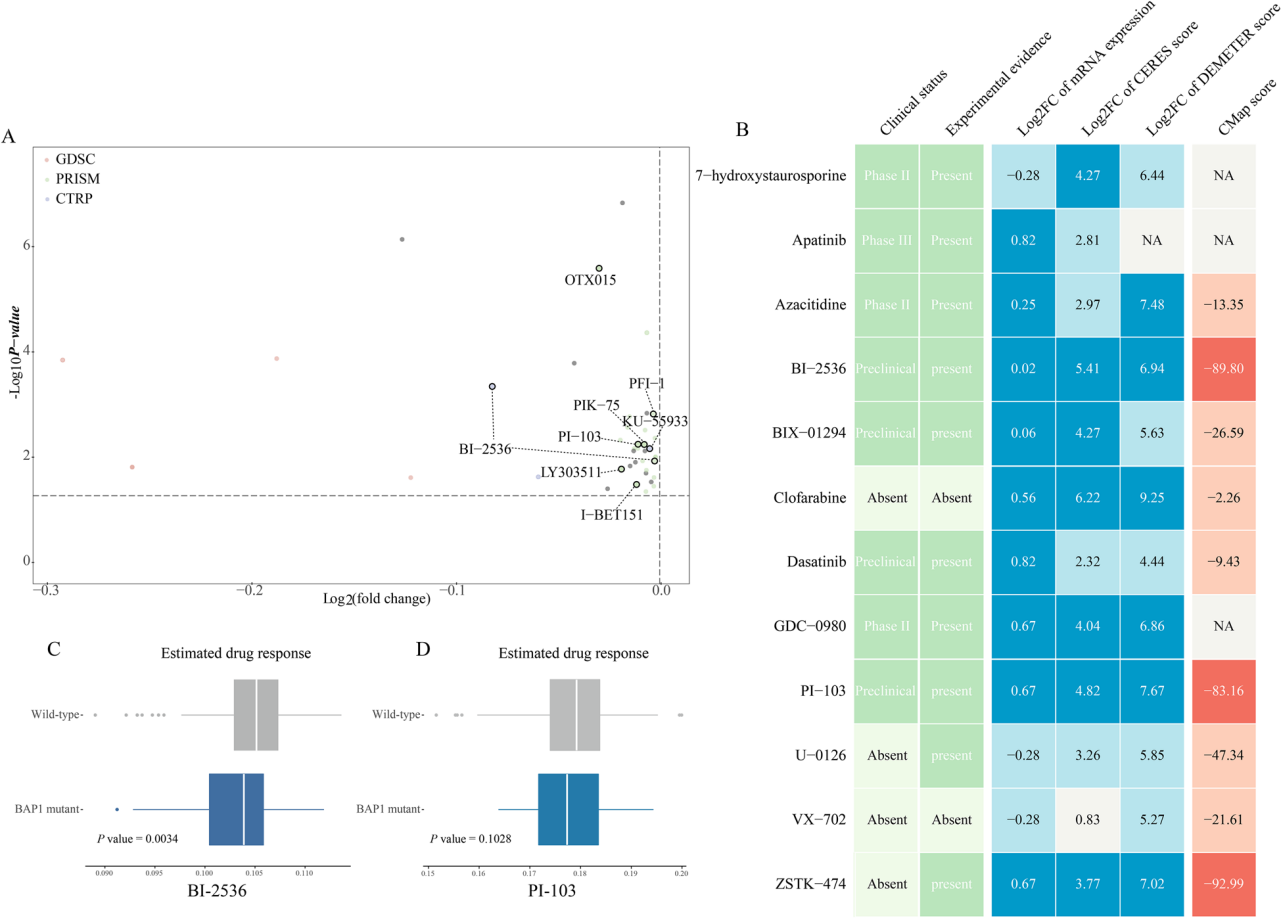
Estimating drug responses of BI-2536 and PI-103 across BAP1 mutated renal cancer patients. (**A**) Differential drug response analyses of identified 26 compounds with potential therapeutic efficacies on *BAP1*-mutant ccRCC. The *BRD4* and *PRKDC* inhibitors with significant response differences between *BAP1* mutant and wild type groups were labeled on the plot. (**B**) Summarizing the current evidences, target gene expression, drug dependency and CMap analysis of candidate drugs. (**C**) Estimating the drug responses of BI-2536 and PI-103 in treating *BAP1* mutated and wild-type RCC patients.

In the DM-DG-drug network, these analyses yielded 26 compounds with potential therapeutic effects for treating *BAP1*-mutant ccRCC. We compared the dependency scores of specific compound targets between the *BAP1* mutant and WT cells from RCC to validate the effect of these potential drugs (Fig. 4C–F). Although there was no statistically difference in results, CCLs with the *BAP1* mutation still exhibited a trend toward the lower dependency scores. Through integrating drug prediction results, survival and dependency analyses, it was found that the *BRD4* and *PRKDC* could be the optimal targets for treating ccRCC patients with the *BAP1* mutations (Fig. 5A). Nevertheless, above analyses alone cannot fully support the conclusion that the actual clinical effect of compounds when used in tumors was consistent with the theoretical inference. Therefore, the multiple perspective approaches for drug prediction were adopted to explore the potential effect of these compounds in treating ccRCC. First, the connectivity map (CMap) analysis was utilized to find candidates whose drug signatures, namely drug-induced profiles of expression changes, were opposite to the *BAP1* mutant expression pattern. A total of three compounds, including ZSTK-474, BI-2536 and PI-103, had CMap scores less than −80, representing the therapeutic efficacy in patients with the *BAP1* mutations. Second, the expression differences of candidate DG were calculated between normal and tumor tissues, and compounds with higher fold change values were considered to have greater potential for ccRCC treatment. Third, through searching relevant literature on these compounds in PubMed (https://pubmed.ncbi.nlm.nih.gov/), we found out the experimental and clinical evidence of candidates in treating ccRCC. Lastly, the dependency analysis of the DGs across kidney CCLs was conducted, and lower CERES or DEMETER scores denoted that the relevant genes were more likely to be essential for the CCLs survival. All results are presented in Fig. 5B and Supplementary Table 7. In general, the BI-2536 and PI-103 that had robust abilities in vitro and in silico, were considered the best therapeutic compounds for the *BAP1* specific ccRCC treatment.

In addition, an independent dataset, which comprised molecular profiles and mutation data of 246 ccRCC patients from the combined microarray cohort, was also used for further external validation. By comparing the estimated AUC values of two specific agents (BI-2536 and PI-103) between the *BAP1* mutant and WT groups, the mutant group was more sensitive to both BI-2536 and PI-103 than the WT group, highly consistent with the results of the in silico prediction (Fig. 5C,D and Supplementary Table 7).

## Discussion

A recently accepted concept of tumorigenesis and progression is that tumor cells are susceptible to mutation events, thus they depend on other genes to gain survival advantages. Considering a pivotal challenge to rescue the activity of driver targets, it is urgent to discover alternative approaches. Fortunately, pharmaceutical agents based on SL strategy provide novel insight for precisely killing tumor cells with certain mutations. The PARP inhibitor Olaparib is the first drug to be clinically used in treating breast cancer patients with *BRCA1/2* mutation based on the SL interaction mechanism^22^. Although pan-cancer analysis has obtained considerable results^10,23^, the practical application value in ccRCC patients may be limited due to their distinct metabolism process, proliferative characteristic and genetic feature.

The applications of RNA interference (RNAi) and clustered regularly interspaced short palindromic repeats (CRISPR) are preferable choices to identify SL pairs, but such methods are expensive and only suitable for screening partners of few fascinating driver genes^8,24^. Currently, given the easily accessible genomic data, using computational procedure is attractive to predict SL pairs. In the current study, we performed SL interaction analysis in the most comprehensive metadata set of ccRCC so far, which included 610 patients from five clinical cohorts with available expression and mutation data, to predict the potential gene pairs. The first predictive method, differential gene expression, assumes that most mutations of driver genes result in loss-of-function and hence allows the tumor cells to compensatively up-regulate the expression of the SL partners^9^. The second predictive method, pairwise gene co-expression, depends on the concept that SL pairs seem to exert related biological functions and co-express in WT tumor samples^9^. The third predictive method, functional similarity, indicates that gene pairs with SL interaction are likely to engage in similar biological process, thus their locations in GO topological network should be neighboring. The last one, mutual exclusivity, is based on the notion that inhibition of two genes with SL interplay can reduce tumor cells vitality and hence two genes of tumor samples express in a mutually exclusive manner^25^.

Classifying the genomic characteristics provides a brilliant prospect for the occurrence, progression and precise treatment of RCC. That is, *VHL* mutation acts as an initiative event to induce tumor occurrence, while *PBRM1*, *BAP1* and *SETD2* cause DNA repair defect and cell overgrowth. Subsequently, the effective pathways, such as PI3K-mTOR activation, confer tumor cells the potential to evade death signals and metastasis^6^. In this study, chromatin remodeling gene *BAP1* accounts for 59.7% of potential SL-based driver genes, followed by another frequent mutating gene *PBRM1* (23.6%). It is revealed that *BAP1* and *PBRM1*, residing closely on chromosome 3p, are frequently mutated (approximately 10% and 40%, respectively) in RCC patients^26–28^. Several studies have proved the crucial role of *BAP1* and *PBRM1* in tumor development. Briefly, BAP1 interacts with BRCA1/ BARD1 complex to regulate crucial biological processes, such as chromatin modification, DNA damage repair and cell cycle control^29,30^. Depletion of *BAP1* was associated with aggressive histological grade^27^, advanced tumor stage^31^ and poor prognosis^29^. Additionally, *BAP1* mutation was correlated with high genome instability index (GII) and low intratumoural heterogeneity (ITH), conferring the adaptive advantage and single lethal target to ccRCC clone^32^. In regards to *PBRM1*, its depletion promoted the upregulation of *HIF-1α*, *STAT3* and the activation of mTOR signaling induced by *VHL* mutation^33^. Such phenomenon may explain that patients with *BAP1* mutation experienced a worse outcome than patients with *PBRM1* mutation after receiving first-line VEGFR inhibitor everolimus and mTOR inhibitor sunitinib treatment^28^. *VHL* represents the most widely mutated gene in ccRCC, and CAMKK1, RORA, and TYK2 were identified as potential SL partners of *VHL* in this study (Fig. 3A, Supplementary Table 3). Among them, JAK kinase TYK2 might be the most promising therapeutic target towards *VHL*-loss ccRCC patients, since the overall survival of TYK2 high expression group was significantly higher than that of TYK2 low expression group in patients with *VHL* mutation. It was reported that *VHL*-mutated RCC cells performed elevated TYK2 activity, while the invasive and metastasis features of *VHL*-mutated cells were reversed by JAK kinase inhibitors^34^. It cannot be ignored that the current computational method involves four rigorous screening criterions, which may lead to some effective SL pairs being ignored since they cannot meet all the requirements.

To explore available compounds for clinical usefulness, we further estimated drug response of clinical samples from pharmacogenomics profile databases CTRP, PRISM and GDSC. The estimated drug sensitivity of bromodomain containing 4 (BRD4) inhibitor BI-2536, phosphoinosmde-3-kinase (PI3K)/mammalian target of rapamycin (mTOR) inhibitor PI-103, and PI3K specific inhibitor ZSTK474 in *BAP1* mutated samples are attractive for further study due to their desirable matching scores. In this study, BI-2536 showed a high drug sensitivity against *BAP1* mutated samples by inhibiting BRD4 function. Among ccRCC patients with *BAP1* mutation, the up-regulated expression of *BRD4* was associated with poor prognosis, indicating a possible benefit of BRD4 inhibition in *BAP1* mutated samples. It is well-known that BRD4, an important component of the bromodomain and extra terminal (BET) protein family, shares similar functions with BAP1 in chromatin remodeling and transcriptional regulation^35,36^. The up-regulation of *BRD4* expression was found in RCC tissues, and associated with advanced histological stage and lymph node metastasis, while knockdown of *BRD4* reduced cell vitality and inhibited tumor growth^37^. The BRD4 inhibitor JQ-1 enhanced the anti-tumor activity of the mTOR inhibitor Palomid 529 in RCC cells^38^. Malignant peripheral nerve sheath tumor with *PRC2* loss-of-function was sensitive to BRD4 inhibitor, suggesting a promising therapeutic approach of SL-based BRD4 inhibition^39^. The dual PI3K/mTOR inhibitor PI-103 is available to treat various tumor types. For example, the inhibitory ability of SCD-1 interference on cell proliferation and migration of RCC cells was amplified by PI-103^40^. Combination of PI-103 and mTOR inhibitor rapamycin performed a better therapeutic effect than single agents in human ovarian and prostate cancer cells, and can effectively prevent rebound activation of the Akt pathway after rapamycin treatment^41^. In addition to the PI-103, ZSTK474, another inhibitor that specifically targets PI3K, also received a high score in our analysis. In vitro experiments have shown that it can inhibit the proliferation of tumor cells through interfering cell G0/1 stage arrest^42,43^. It is exciting that ZSTK474 induced the degradation of multidrug efflux pumps ABCB1 and ABCG2 so as not to be affected by the efflux effect of resistant cancer cells^44^. Furthermore, ZSTK474 exhibited antiangiogenic activity via downregulating HIF-1α and VEGF, and suppressed renal cancer growth in a xenograft model^45^. Generally, above evidences of these three compounds indirectly proved the reasonability of our computational pipeline and the reliability of the prediction results.

This study still has several limitations. First, several studies employed pairwise survival analysis to SL identification^9,46^, which was not included in our screening criteria, for the reason that the relatively low mutation frequency of crucial driver genes like *BAP1* and some inaccessible survival data of cohorts would reduce the statistical power and thus ignore several important SL interactions. Second, despite the robust evidence from pharmaceutical database, there is still a lack of experimental validation. Related experiments are needed in the future to support our conclusions. Third, BI-2536 is also considered as PLK1 inhibitor^47^, so further exploration of the target of BI-2536 is essential to elucidate its anti-cancer mechanism in ccRCC.

In conclusion, capitalizing on extensive screening data combined with molecular and clinical data from multiple cohorts, this study developed a novel computational-based strategy to identify SL pairs for ccRCC patients harboring genetically mutation as well as some potential therapeutic agents for *BAP1* mutated patients. The potential SL-associated partners for *BAP1* and *PBRM1*, two frequent altered genes, have complemented the current *VHL*-predominant research and mapped a comprehensive landscape for SL interaction in ccRCC, which might help to deepen our understanding of ccRCC mutation patterns and provide an alternative strategy of personalized renal cancer treatment.

## Materials and methods

### RNA-sequencing cohorts

In total, five RNA-sequencing cohorts of ccRCC, including TCGA-KIRC cohort^11^, RECA-EU, CM-009^12^, CM-010^13^ and CM-025^14^ were used in this study. Of these, gene-expression, mutation profiles and full clinical annotations of TCGA-KIRC, RECA-EU were obtained from the Cancer Genome Atlas (TCGA) database (https://portal.gdc.cancer.gov/repository) and the International Cancer Genome Consortium (ICGC) portal (https://dcc.icgc.org/). The relevant information about CM cohorts was achieved from the supplementary files of three prospective clinical trials which comprised of ccRCC patients treated with anti-PD-1 antibody immunotherapy^48^. All expression data (raw counts) of RNA-sequencing datasets mentioned above were transformed into transcripts per million (TPM) values and these RNA-seq cohorts were integrated into one combined metadata. The ComBat algorithm of *SVA* R package^49^ was applied to correct batch effects from non-biological technical biases to ensure comparability between different cohorts (Supplementary Fig. 4A). The single nucleotide variants (SNVs) and small insertions/deletions (INDELs) of mutation data were saved for further analysis, while copy number variants (CNVs) profiles were not included due to the data limitation. In order to evaluate the effect of mutation on gene expression, the expression data and functional mutations were involved in this study. Notably, functional mutations, including frameshift and nonsense mutations, were defined as alternations that the resulting proteins usually affected normal physiological functions of cells. The non-functional mutations, including silent mutations (synonymous mutations) were excluded and samples with no functional mutations or fewer than ten mutations in gene panels were considered as outliers and discarded from downstream analyses. Genes with duplicated mutations were merged to keep only one record.

### Microarray cohorts

The expression data, somatic mutations data and clinical information of E-MTAB-1980^50^ (including 101 ccRCC samples based on GPL13497), E-MTAB-3218^51^ (including 114 ccRCC samples based on GPL13667), E-MTAB-3267^52^ (including 59 ccRCC samples based on GPL6244) were acquired from the ArrayExpress database (https://www.ebi.ac.uk/arrayexpress/). Then background adjustment and quantile normalization were performed on these raw expression files from Affymetrix and Agilent by using the robust multiarray average (RMA) method located in R package *Affy*^53^. For GSE29609 cohort^54^ (including 39 ccRCC samples based on GPL1708), the expression data and detailed clinical information were collected from the Gene Expression Omnibus (GEO) (http://www.ncbi.nlm.nih.gov/geo/) and the raw expression data were also normalized by the RMA method. These microarray cohorts were merged into one combined cohort with batch effect removal using ComBat function (Supplementary Fig. 4B). Additionally, the mutation annotation information of GSE29609 cohort is unavailable.

### Cancer cell line data

Gene expression profiles and somatic mutation data of human CCLs were downloaded from the Cancer Cell Line Encyclopedia (CCLE) project (https://portals.broadinstitute.org/ccle/) and Genomics of Drug Sensitivity in Cancer (GDSC) project (https://www.cancerrxgene.org/). The experimental information of different drug responses against CCLs was achieved from the Cancer Therapeutics Response Portal (CTRP v2.0, released October 2015, https://portals.broadinstitute.org/ctrp), PRISM Repurposing dataset (19Q4, released December 2019, https://depmap.org/portal/prism/) and GDSC 1&2 datasets (Release 8.2, release February 2020, https://www.cancerrxgene.org/downloads/bulk_download), respectively. Of these medicine databases, PRISM contained the drug sensitivity data of 1448 compounds against 499 CCLs, CTRP provided the drug sensitivity data of 545 compounds against 907 CCLs, and GDSC included the drug sensitivity data of 518 compounds against 988 CCLs. And the AUC values of dose–response acquired from these three datasets were used as evaluation indicators of drug sensitivity, which lower AUC value suggests higher response probability to therapy treatment. Compounds with missing AUC values across more than 20% of the CCLs were excluded firstly, and the rest of compounds containing incomplete data were imputed using the K-nearest neighbors (KNN) method^55^ located in R package *Impute*. Notably, expression profiles and molecular data of CCLs were downloaded from the same CCLE Project, and were used for subsequent PRISM and CTRP analyses. In order to investigate the cancer survival-essential genes, the genome-wide gene dependency scores, including CERES scores from clustered regularly interspaced short palindromic repeats (CRISPR) knockout screens^56^ and DEMETER scores from RNA interference (RNAi) screens^57^, were achieved from the Cancer Dependency Map (DepMap) portal (https://depmap.org/portal/download/), which lower CERES or DEMETER scores denote that relevant genes are more likely to be essential in cell survival and proliferation of CCLs.

### *BAP1* mutation prediction

Due to missing mutation data of part samples in E-MTAB-3267, E-MTAB-3218 and GSE29609, EN-based prediction model, a generalized linear model in the R package *glmnet*^58^, was utilized to forecast *BAP1* mutation status. The RNA-seq metadata mentioned above were then used as training cohort to construct the prediction model, and samples with mutation annotations in E-MTAB-1980 were considered as external validation for evaluating the performance of *BAP1* prediction model. To select significant genes which were taken as input into EN model (abs (Log2FC) > 1.5 & adjust *P* < 0.05), differential expression analysis between the *BAP1* mutant and WT samples from the training cohort was performed using the R package *limma*^59^. Additionally, the leave-one-study-out cross-validation was performed to evaluate the accuracy of EN model. Specifically, after splitting a dataset into a training set and a testing set, and using all but one observation as part of the training set, the prediction model was built using data from the training set. Lastly, this process was repeated *n* times (where *n* is the total number of observations in the dataset), leaving out a different observation from the training set each time, which meant that it provided a much less biased measure of test mean squared error compared to other cross-validation methods. Notably, the penalty was set as 0.9 in fitting a generalized linear model. The predictive performance of the EN model in training and validation cohorts was evaluated using ROC curve via the R package *pROC*^60^.

### Detection of cancer driver mutations

To discern likely DMs regulating gene network of tumor expression from thousands of mutations, the *DriverNet* algorithm^61^ was applied in this study, which could evaluate the DM probability through integrating genome and expression data. Accordingly, a mutation matrix, a corresponding expression matrix and an influence graph were taken as input documents of *DriverNet*. In this analysis, the influence graph was derived from the Reactome Functional interactions^62^, an updated protein functional interaction network (Version 2020). Notably, the results of *DriverNet* indicated the probabilities whether imported mutations belong to DMs, and genes with *P* value < 0.05 were deemed statistically significant. To make our prediction more reliable, we compiled a comprehensive list of cancer-associated driver genes which have been validated from prior studies and made a comparison between our prediction and previous results. These same DMs were saved for constructing the network between DMs and DGs subsequently.

### Collection of drug-target interactions

The medicine information about drug-target was acquired from the Drug Repurposing Hub^63^ and DrugBank^64^, respectively. The Drug Repurposing Hub (released March 2020, https://clue.io/repurposing#download-data) contained 6798 unique compounds and 2183 targeted genes, and DrugBank (Version 5.1.8, released January 2021, https://go.drugbank.com/releases/latest) comprised 7540 compounds and corresponding 3976 targeted genes. Then two drug data were merged into one meta-drug set, and a total of 11,875 compounds and 4465 DGs were identified after removing duplicated medicine information. In order to identify genes with potential therapeutic implications, DGs were utilized to construct DM-DG-drug network.

### Mutual exclusivity analysis

Under the SL hypothesis, no somatic alteration happens on both genes of candidate partners in ccRCC simultaneously. Based on the somatic mutation data of 1211 patients, the analysis was performed by using the *DISCOVER* R package to determine significant mutual exclusivity^65^. Gene pairs with *P adjust* value < 0.1 were considered statistically significant.

### Connectivity map analysis

To identify potentially therapeutic compounds, CMap analysis (https://clue.io/) was used for searching compounds of which gene expression patterns were opposite to the *BAP1* mutant expression pattern. Differential analysis between *BAP1* mutant and WT samples was performed to select 150 up-regulated and down-regulated genes with the most significant fold changes respectively. Through the CMap analysis, the standardized connectivity score for each perturbation was calculated, which ranges from −100 to 100. Compounds with the CMap score < −80 were considered to have a potential therapeutic effect for ccRCC.

### Identification of ccRCC subclasses

NBS was performed to identify subclasses of ccRCC via Python package *pyNBS*^66^, which divides tumor samples with available somatic mutation profiles into molecularly and clinically relevant subtypes on the basis of the mutation characters of the combined RNA-seq cohort^67^. Through integrating a high-quality cancer reference network from the recent study^66^ and a mutation matrix of driver genes, we acquired the resulting data which contained the clustering information and corresponding consensus matrix from NBS. To evaluate the robustness of clusters *k* ranging from 2 to 5, the cophenetic correlation coefficient was calculated using the R package *NMF*^68^ and the value of *k* with the maximum cophenetic correlation coefficient was considered as the optimal number of clusters. In addition, the nearest template prediction (NTP) analysis was conducted via R package *CMScaller*, which could predict the previously published RCC classifications based on the provided subclass signatures^69^.

### Functional similarity analysis

In this study, *GOSemSim*, an R package for measuring semantic similarity among GO terms and gene products^70^, was utilized to estimate the similarity of MF and CC among different genes. Gene pairs achieved from DM-DG network above were used to measure FSS, which was calculated based on the semantic similarity in MF (SsMF) and CC (SsCC), as following formula:

$$\text{Functional Similarity Score}= \sqrt{SsMF*SsCC}$$

Notably, gene pairs with FSS > 0.45 were considered to have high functional correlations and were used for further analysis.

### Rank aggregation analysis

To obtain a consistent result across multiple sources, *rank aggregation* algorithm, an order statistics-based method located in R package *RobustRankAggreg* proposed by Kolde et al^71^, was applied in this study, of which the result (*P* value) indicates whether the ranking of a particular gene pair is statistically significant. In this analysis, we chose the order statistics method proposed by Stuart et al^72^ by assigning the corresponding parameter to ‘*the Stuart*’ and defined the rank aggregation score (RAS) as follows:

$$Rank\, Aggregation\, Score= -log2(\mathrm{P value})$$

The ranking of candidate gene pairs was determined by the RAS, and a higher RAS denoted a more concordant ranking.

### Predicting drug response in clinical samples

Three large pharmacogenomic datasets, including CTRP, PRISM and GDSC, contained massive drug screening and gene expression data across hundreds of cancer cell lines. Previous studies have demonstrated that drug response in clinical samples can be predicted using data from in vitro cell line experiments^73^. To perform drug response prediction, we intended to test different machine learning methods, including support vector machine, random forest and multivariate linear regression, based on the actual drug sensitivity and molecular data. In this study, the ridge regression model that exhibited great and precise performance in the previous research^74^ was utilized for transcriptome data-based drug response prediction using the R package *pRRophetic*. Through exploiting the expression and drug response data of solid CCLs from CCLE and GDSC projects (excluding hematopoietic and lymphoid tissue-derived CCLs), this predictive model was trained with a satisfied predictive accuracy evaluated by default tenfold cross-validation and then applied to calculate different drug response across clinical samples. These compounds with positive response calculated by this model were matched to their DGs for subsequent construction of the DM-DG-drug network.

### Enrichment analysis

We performed gene set variation analysis (GSVA) using the R package *GSVA* based on the hallmark definitions (h.all.v7.4.symbols) extracted from the Molecular Signatures Database (https://www.gsea-msigdb.org/gsea/msigdb/)^75^ to explore the differential expression of certain pathway or signature between *BAP1* and WT patients^76^. Notably, the resulting *P value* from the hypergeometric test was adjusted for multiple comparison testing and *P adjust* < 0.05 was considered significant.

### Statistical analysis

All the statistical tests and graphical visualization were conducted utilizing R statistical software, version 4.0.5 (https://cran.r-project.org/). Student’s t-test or Wilcoxon rank-sum test was applied for comparison of two groups with or without normally distributed variables, respectively. Similarly, correlation between two continuous variables was measured by either Pearson’s r correlation (measure of linear relationship between two continuous variables) or Spearman’s rank-order correlation (nonparametric measure of statistical dependence between two variables). Contingency table variables were analyzed by Fisher’s exact tests. The Kaplan–Meier method was applied to perform survival analysis and the statistical significance of differences was determined using the log-rank (Mantel-Cox) test. The hazard ratios (HR) were calculated using the univariate Cox proportional hazards regression model located in R package *survival*. The Benjamini–Hochberg method was utilized to adjust *P* value of multiple testing in those analyses with more than 20 comparisons. *P* value < 0.05 was considered statistically significant for all computational analysis unless otherwise stated.

## Supplementary Information

Supplementary Figures.

Supplementary Tables.

## Data Availability

All data used in this study are publicly available. The TCGA-KIRC dataset is available in the Cancer Genome Atlas (TCGA) database (https://portal.gdc.cancer.gov/repository). The RECA-EU dataset is available in the International Cancer Genome Consortium (ICGC) portal (https://dcc.icgc.org/). The E-MTAB-1980, E-MTAB-3218, E-MTAB-3267 datasets are available in the ArrayExpress database (https://www.ebi.ac.uk/arrayexpress/). The GSE29609 set is available in the Gene Expression Omnibus (GEO) (http://www.ncbi.nlm.nih.gov/geo/). The Human cancer cell lines data are available in the Cancer Cell Line Encyclopedia (CCLE) project (https://portals.broadinstitute.org/ccle/) and Genomics of Drug Sensitivity in Cancer (GDSC) project (https://www.cancerrxgene.org/). The drug responses data are available in The Cancer Therapeutics Response Portal (https://portals.broadinstitute.org/ctrp), PRISM Repurposing dataset (https://depmap.org/portal/prism/) and GDSC 1&2 datasets (https://www.cancerrxgene.org/downloads/bulk_download). The drug-target data were available in The Drug Repurposing Hub (released March 2020, https://clue.io/repurposing#download-data) and DrugBank (Version 5.1.8, released January 2021, https://go.drugbank.com/releases/latest). All codes required to reproduce the results were available from the first author upon reasonable request.
