# Supplementary material for: Exploring synthetic lethal network for the precision treatment of clear cell renal cell carcinoma
Source: Sci Rep. 2022 Aug 2;12:13222. doi: 10.1038/s41598-022-16657-7 (PMC9345903; doi:10.1038/s41598-022-16657-7)
Supplement: Supplementary file 1 — Supplementary Figures. [file 41598_2022_16657_MOESM1_ESM.pdf]

Supplementary Information

The PDF file includes:

Supplementary Figure 1. Classification of clinical samples based on mutation data and difference of tumor mutational burden (TMB) between NBS1 and NBS2.

Supplementary Figure 2. Survival difference between *BAP1* mutant and WT groups.

Supplementary Figure 3. Evaluation of the performance of prediction model.

Supplementary Figure 4. Evaluation of the results of batch effect correction.

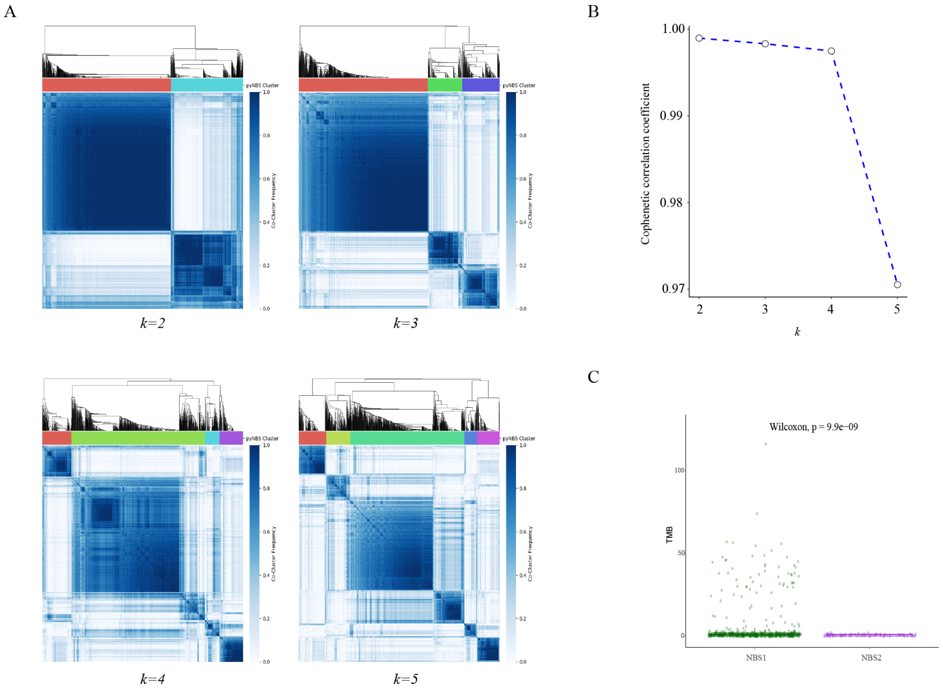

Supplementary Figure 1. Classification of clinical samples based on mutation data and difference of tumor mutational burden (TMB) between NBS1 and NBS2 (A) Consensus matrix of network-based stratification (NBS) based on mutation data for k=2-5. (B) Cophenetic correlation coefficient under corresponding k values. (C) Difference of tumor mutational burden (TMB) between NBS1 and NBS2

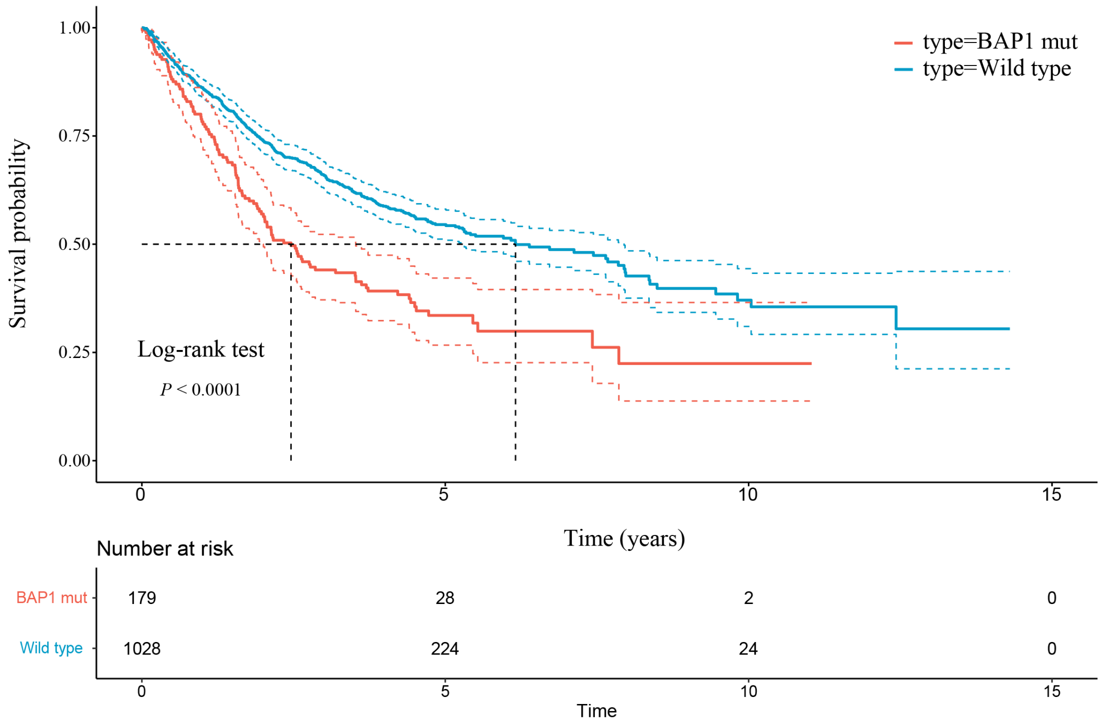

Supplementary Figure 2. Survival difference between *BAP1* mutant and WT groups.

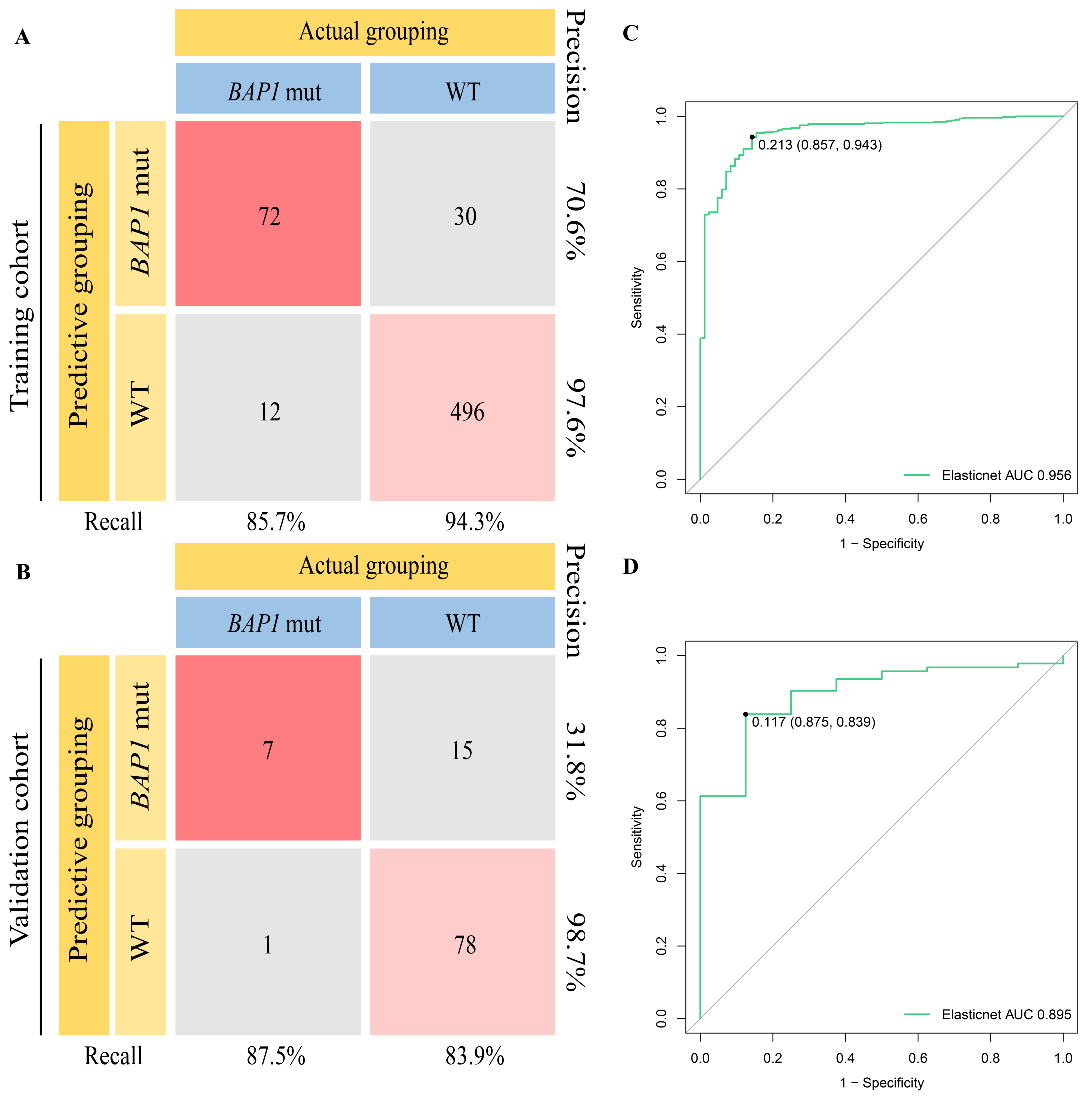

Supplementary Figure 3. Evaluation of the performance of prediction model. (A-B) The confusion matrix of the prediction results in the training cohort and the validation cohort. (C-D) The receiver operating characteristic (ROC) curve of the prediction results in the training cohort and the validation cohort. AUC values close to 1 indicate a high true positive rate with low false positive rate.

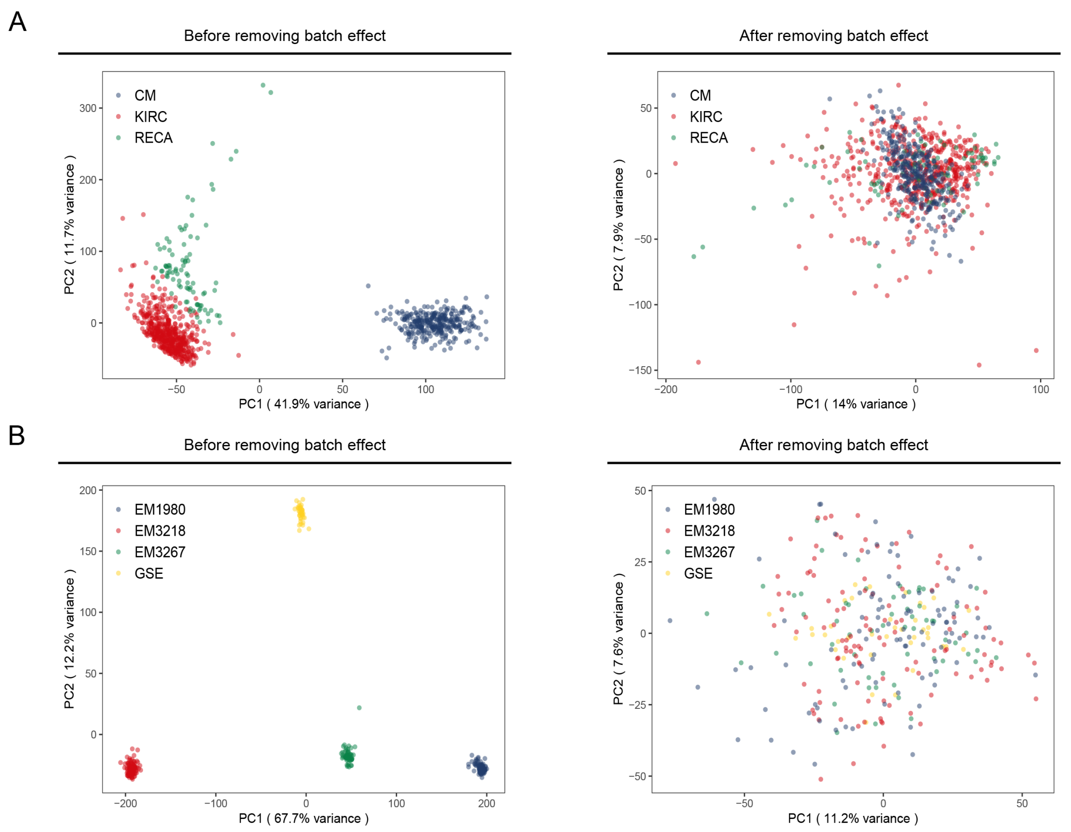

Supplementary Figure 4. Evaluation of the results of batch effect correction. (A)The principal component analysis (PCA) before (left) and after (right) batch effect correction on RNA-seq cohorts. (B)The principal component analysis (PCA) before (left) and after (right) batch effect correction on Microarray cohorts.
